# Situation analysis of evidence-informed health decision-making in Lao PDR: the case of health technology assessment

**DOI:** 10.1016/j.lanwpc.2025.101534

**Published:** 2025-04-09

**Authors:** Sysavanh Phommachanh, Manit Sittimart, Aparna Ananthakrishnan, Souphaphone Vongsack, Soudavanh Soysouvanh, Elizabeth A. Ashley, Yot Teerawattananon, Saudamini Vishwanath Dabak, Mayfong Mayxay

**Affiliations:** a Unit for Health Evidence and Policy, Institute for Research and Education Development, University of Health Sciences, Lao PDR; b Health Intervention and Technology Assessment Program Foundation (HITAP), Nonthaburi, Thailand; c Lao-Oxford-Mahosot Hospital-Wellcome Trust Research Unit, Microbiology Laboratory, Mahosot Hospital, Vientiane, Lao PDR; d Centre for Tropical Medicine and Global Health, Nuffield Department of Clinical Medicine, University of Oxford, Oxford, United Kingdom; e Saw Swee Hock School of Public Health, National University of Singapore, Singapore

**Keywords:** Lao PDR, Health policy, HTA, Evidence-informed decision making, Priority setting

## Abstract

**Background:**

There is increasing interest in using evidence to inform policy in Lao PDR, which is in the process of establishing a unit for Health Technology Assessment (HTA). This situation analysis aims to explore the current landscape of evidence generation and translation into policy decisions in Lao PDR using the case of HTA.

**Methods:**

A mixed methods approach was applied. Self-administered questionnaires and semi-structured interviews were conducted with different stakeholder groups. Data were analysed thematically and summarised in tabular form.

**Findings:**

There were 212 responses to the survey and 38 stakeholders were interviewed between March and September 2021. The health policy decision process in Lao PDR is based on consultation meetings, influenced by external experts and/or companies, without consistent use of evidence. There remains a lack of human resource and infrastructure for health evidence to inform policy. Two-thirds of the respondents to the survey strongly agreed that HTA helps in efficient allocation of health resources and improving quality of healthcare. Half of the respondents perceived that HTA can impact the government budget and transparency, which was consistent with findings from the qualitative data. Use of economic considerations was limited in Lao PDR. HTA was seen to apply to policy areas, notably for reimbursement. Only a few organisations can supply health evidence and HTA output, and more training and multi-disciplinary collaboration is needed to conduct and produce HTA and other health evidence to inform policy in Lao PDR. Funding for HTA remains a concern.

**Interpretation:**

Improvement of the health policy decision process is urgently needed in Lao PDR. Limited capacity to conduct HTA as well as institutional considerations need to be addressed. Recent efforts towards this end through the establishment of a unit focused on HTA, capacity building activities and international collaborations are promising to establish evidence-informed priority setting for health policy and can also benefit from regional efforts in this direction. This type of approach to assess the situation for evidence use will be beneficial for other countries embarking on this path.

**Funding:**

10.13039/100010269Wellcome Trust, the United Kingdom 10.13039/501100000276Department of Health and Social Care (DHSC), and the 10.13039/501100004397Ministry of Public Health, Thailand.

**Research in context**

**Evidence before this study**

Lao PDR, a lower-middle income country situated in Southeast Asia, is transitioning from donor support for its health systems and has been planning to implement Health Technology Assessment (HTA) to inform priority setting for health policies and ensure its sustainability. Previous research has shown that there is a need for increasing domestic research capacity and strengthening communication between researchers and policymakers as well as highlighting the importance of international collaborations.

**Added value of this study**

To our knowledge, this is the first study providing an overview of the current and potential use of HTA for health policy decision-making in Lao PDR.

**Implications of all the available evidence**

Given limited resources available in the country, including the transition of Lao PDR from Gavi support, the increasing competition for resources for different national priorities within a limited fiscal space, and more broadly, regional efforts to strengthen HTA, there is an urgent need to invest in building technical and institutional capacity for using HTA as a tool for priority setting in Lao PDR and strengthen international collaborations. The establishment of the Unit for Health Evidence and Policy (UHEP) for generating evidence and defining processes for its use is promising for the future.

## Introduction

Globally, countries have made a commitment to achieve Universal Health Coverage (UHC) by 2030, and ensure affordable and high-quality essential health services for all.^1^ However, given limited resources, policy makers must make choices in terms of which interventions to provide. Health Technology Assessment (HTA), a multidisciplinary approach to inform decisions in health that takes into account economic and other considerations, has been identified as a means to support sustainability of UHC.^2^^,^^3^ Over the past decade, many countries, including low- and middle-income countries (LMICs) have been increasingly adopting HTA to facilitate resource allocation in an evidence-informed manner.^4^, ^5^, ^6^, ^7^

HTA has a technical component using, for example, economic evaluations to demonstrate the cost-effectiveness of health interventions. This technical component is embedded in a process that begins from topic nomination and selection, to assessment, appraisal, dissemination of recommendations, implementation and evaluation.^8^ An important application of the role of HTA-informed decisions is in assessing health products and services for inclusion into national health insurance schemes, also known as health benefit packages.^9^ For HTA decisions to be successful, infrastructure to support data collection, analysis, and evidence appraisal by relevant stakeholders are fundamental.^10^ Legal and governance frameworks for using evidence in health decision-making have been created in some countries, however, the capacity for conducting and using evidence varies across countries. In recent years, there has been a movement to strengthen HTA capacity in the World Health Organization Southeast Asia Region (WHO SEAR), members of which work closely with other countries in Southeast Asia that are in the WHO Western Pacific (WHO WPRO) and to harmonise HTA among the member nations of the Association of Southeast Asian Nations (ASEAN). These initiatives seek to address the limited capacity and also reduce duplication of efforts.^11^, ^12^, ^13^

The Lao Peoples’ Democratic Republic (PDR), a land-locked country in Southeast Asia, has been exploring the application of HTA for health policy decisions.^14^ Classified as a lower middle-income country, Lao PDR has witnessed rapid development over the last two decades, with steady gains in lowering poverty levels and improving health outcomes such as infant, maternal mortality and life expectancy.^15^ However, these developmental gains were significantly impacted during the COVID-19 pandemic in addition to a shrinking economy. Lao PDR will be transitioning from Gavi support in the near future, requiring the country to identify sustainable solutions for funding its vaccination programmes moving forward.^16^^,^^17^ These factors, alongside increasing competing priorities of the country, warrant a more judicious use of resources to support rising healthcare expenditure, and a system that will offer evidence-informed means of reallocating existing resources to provide for essential care.

In Lao PDR, there is limited literature on the use of evidence for health decision making and HTA. To this end, this study was conceptualised to explore the current landscape of evidence generation and translation into policy decisions using the case of HTA. The study focuses on HTA given the interest to institutionalise HTA by establishing the Unit of Health Evidence and Policy (UHEP), the first HTA body in the country, at the University of Health Sciences (UHS), with support from the Wellcome Trust and the UK Department of Health and Social Care (DHSC). Additionally, there is regional commitment to build capacity for HTA and the research teams involved in this study have experience in conducting HTA. The study therefore examines the need, demand and supply of HTA as well as current practices of health policy decision-making in Lao PDR and provides context and discusses challenges and opportunities for strengthening the use of evidence in the country.

## Methods

### Study objectives

The primary objective of this study was to conduct a situation analysis of the use of evidence, specifically HTA for policy in Lao PDR. Specific objectives are:

1. To investigate the current approaches employed in using HTA for policy making within national and provincial systems and the need for HTA
2. To understand the demand for HTA
3. To understand the supply of HTA and map relevant stakeholders
4. To recommend potential areas for application of HTA in Lao PDR

### Conceptual framework and research tools

**Fig. 1:**
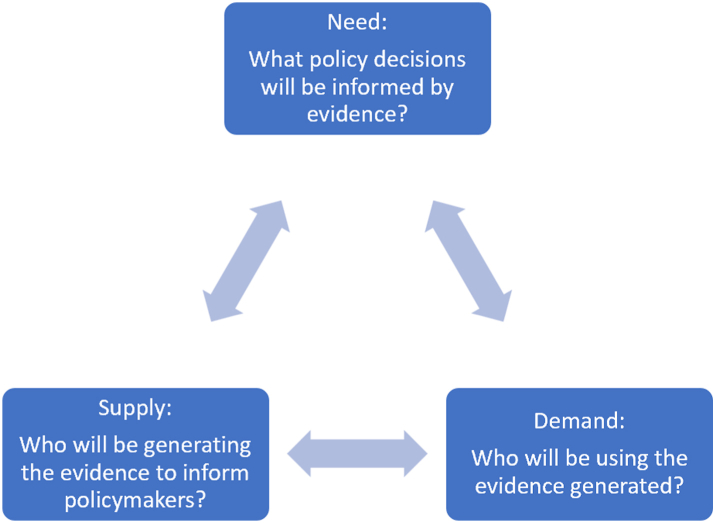
Conceptual framework.

This study used the conceptual framework depicted in Fig. 1. This conceptual framework has been adapted from a framework developed by the International Decision Support Initiative (iDSI).^18^ The framework used for this study comprises three parts: the need, demand and supply for evidence, using HTA and other health service research. The “need” refers to the health policy decisions requiring HTA or other evidence that need to be made for example, deciding on developing health benefits packages for reimbursement or clinical practice guidelines. It may also refer to attributes of decision-making such as efficiency, transparency and equity that are important to stakeholders in a country as well as the policy areas such as maternal and child health or health interventions or technologies that are required (e.g., medicines or health promotion activities). The “demand” refers to the current or potential users of HTA or evidence generated, and could include entities in a health system responsible for developing policies or payers for a public health insurance scheme. The “supply” refers to those entities in the health system that generate evidence as well as the availability of inputs required to generate the evidence (e.g., cost data or information on health data). The challenges and opportunities for utilising HTA or other relevant evidence for policy are also important to examine. The framework was used to design the questionnaires for the Self-administered survey and Semi-structured interviews (SSIs) and to identify themes for analysis.

This framework and the approach have been used by members of the research team in other countries to inform the development of HTA.^7^^,^^19^^,^^20^ It has been found to be beneficial in understanding the current practices, actors and infrastructure in the health system that are currently involved or could be involved in HTA in the country. It also has a simple structure that is easy to communicate with diverse stakeholders. Further, its application to the context in Lao PDR will enable comparison with other countries as well. We are not aware of other frameworks used for conducting a situation analysis of HTA or other relevant evidence for health policy. However, we recognise that there are other frameworks that link knowledge translation and the use of evidence for health policy making and also involve understanding the political economy of a health system, which is not considered in the scope of this paper.^21^^,^^22^

### Study design

To address the components of the framework, a mixed methods approach was applied to this study. First, a Self-administered survey, which included open and closed-ended questions, was administered. The survey was made available at the offices of the respondents and online. Thereafter, we conducted Semi-structured interviews with key informants from these different stakeholder groups for an in-depth exploration of selected issues surrounding the health policy context in Lao PDR. The quantitative approach for the survey was undertaken to be able to incorporate the perspectives of respondents representing a range of stakeholders and was followed by in-depth interviews to gain deeper insights from a targeted group where there were gaps in understanding. The linkages between the objectives, conceptual framework and methods applied are presented in Table 1.

**Table 1:** Objectives, conceptual framework and methods.

Objective	Component of the framework	Methods
1. To investigate the current approaches employed in using HTA for policy making within national and provincial systems and the need for HTA	Need	Self-administered survey Semi-structured interviews
2. To understand the demand for HTA	Demand	Self-administered survey Semi-structured interviews
3. To understand the supply of HTA and map relevant stakeholders	Supply	Survey Semi-structured interviews
4. To recommend potential areas for application of HTA in Lao PDR	N/A	Synthesis of results from survey and Semi-structured interviews

### Study participants

Data collection with multiple stakeholders such as Ministry of Health, international organisations, hospitals, educational institutes, research institutes and embassies was purposively carried out. The main focus of data collection for the situation analysis was in Vientiane where policy makers, researchers, and development partners are located. In addition, a few representatives from the provincial health offices in the Northern and Southern part of Laos as well as some participants from outside Lao PDR were invited to participate in the survey and interviews. Participants from outside Lao PDR were included in the sample given previous or on-going collaborations in the health sector. While these organisations may not have a direct influence on the decision-making process, they are involved in providing technical assistance or funding support for activities and have an understanding of the health system in Lao PDR. Participants from outside the capital were included to gain a wider perspective on health decision-making. Stakeholders were classified into three categories as shown in Table 2.

**Table 2:** List of stakeholders for data collection.

Category	Organisations
Demand for health evidence	Policy makers consisting of Ministers of MOH, Directors of concerned departments of MOH (Finance, Planning and Cooperation, Office of Social Insurance, Food and Drug, Personnel, Health Professional Education, Hygiene and Health Promotion, CDC, Health Care and Rehabilitation, and Cabinet), Centers (MCH center, Nutrition Center), Directors of hospitals (Mahosot Hospital, Sethathirath Hospital, MCH Hospital, Mittaphap Hospital, Child Hospital), three provincial health offices, National Immunisation Technical Advisory Group (NITAG), Ministry of Finance, National Assembly.
Supply of health evidence	Supply side: University of Health Sciences (UHS), Lao Tropical and Public Health Institute (TPHI), and other research institutes such as, Lao-Oxford-Mahosot Hospital-Wellcome Trust Research Unit (LOMWRU), Institut Pasteur du Laos (IPL), Centre Christophe Mérieux, Institut De Recherche pour le Development (IRD), Institute of Research and Education Development (IRED), Population Services International (PSI), Swiss Tropical Institute, and National Animal Health Laboratory and Ministry of Agriculture in Lao PDR
Support for processes for use of health evidence	International organisations within Lao PDR and outside: 1) Outside Lao PDR: HITAP, Mahidol Oxford Tropical Medicine Research Unit (MORU), Centre for Global Development (CGD), University of Oxford; 2) Within Lao PDR, we included World Health Organization (WHO), United Nations Population Fund (UNFPA), United Nations Children's Fund (UNICEF), United Nations Development Programme (UNDP), United Nations Office for Project Services (UNOPS), World Bank, Asian Development Bank (ADB), Clinton Health Access Initiative (CHAI), Global Vaccine Alliance (Gavi), LOMWRU, Institut Pasteur du Laos, Centre Christophe Mérieux, Institut de Recherche pour le Développement (IRD), Population Services International (PSI), Japan International Cooperation Agency (JICA).

### Questionnaire development

The questions for the self-administered survey was divided into four sections to address the three components of the framework, namely, the need, demand and supply for HTA: priority setting of needs, identifying the demand, and exploring the supply of HTA and other health research areas or other health service research. Specially, respondents were asked to reflect on their organisation's potential role in HTA. For the semi-structured interviews, three sections covering health policy in Lao PDR, the need demand, and supply of HTA and other health research evidence in Lao PDR, Strength, Weaknesses, Opportunities and Threats (SWOT) of establishing an HTA unit in Lao PDR were included. The questions on the “need” or priorities for HTA allows understanding of the policy questions that HTA could address, while the section on demand seeks to unpack who the users of HTA are or could be and the section on supply seeks to understand the capacity and infrastructure available to respond to the needs and demand. Gaining these perspectives allows an understanding of existing practices and the potential for implementing HTA in the country.

A draft questionnaire was internally tested among organisational staff, including individuals who might or might not have prior experience in HTA, to assess its flow and clarity. Following that, the questionnaire was revised iteratively (see Appendix 1 for the survey questionnaire and Appendix 2 for the interview guideline).

### Data collection

A hybrid training of data collectors was conducted. Data collected was reviewed and team meetings were conducted as needed for clarifications. Data was collected between March and September 2021, during the COVID-19 pandemic, when travel restrictions were in place.

#### Self-administered survey

Online surveys were administered to 350 participants and when needed, questionnaires were also sent as hard copies to participants’ offices. Before conducting the survey, the research team sent an invitation letter, information sheet, and consent form to eligible participants at their offices. The definition of HTA was provided at the start to ensure a common understanding of the term. Refer to Appendix 1 for the survey questionnaire in English.

#### Semi-structured interviews (SSIs)

Semi-structured interviews (SSIs) were conducted face-to-face and online. The interview guideline included questions on the current state of decision-making on health, context of the same from the past and proposals for the future. We also requested examples to support the responses, as these would add more contextual, socio-economic understanding to the subject. The interview guideline is included in Appendix 2. Before the interview started, the interviewers explained the aim of the study and the general topics that would be discussed, including confirmation that the records would be anonymised. Participants from international organisations were interviewed in English by SP, and other Lao participants were interviewed in Lao language by the research team. Two persons conducted each interview, with one person serving as the interviewer and another as the notetaker, who took notes during the interview and later prepared a summary. All interviews were recorded. The time of interviews ranged from 45 to 115 min (average 68).

### Data analysis

Both qualitative and quantitative data were analysed. Firstly, quantitative data were entered into Microsoft Excel (2011) and cleaned; IBM SPSS Statistics 25 was used for analysis. Summary statistics were used to describe the characteristics of participants, their opinions on the policy decision making process in Lao PDR, the need and demand for and application of HTA in the country and are included in the text. Responses to open-ended questions were coded into categories. Secondly, the recordings and interview notes were used to generate verbatim transcripts for data analysis, which were analysed manually. At least two team members independently analysed the interview transcripts using Excel to organise the raw data. A six-step reflexive thematic analysis, as outlined by Braun and Clarke and further elaborated by Byrne, was employed to guide the process.^23^^,^^24^ This included: (i) data familiarisation, (ii) generating initial codes, (iii) identifying themes, (iv) reviewing potential themes, (v) defining and naming themes, and (vi) synthesising findings. The research team engaged in iterative discussions for the open coding process to refine and agree upon the final themes. Themes were considered significant where there was consistency across and within study participants and/or where they deepened understanding and captured something important in relation to the research question. The results from thematic analysis were presented alongside the quantitative findings as part of the data triangulation process.^25^ Responses to closed ended responses to the survey are provided in Appendix 3.

### Ethics

This study received ethical approval from the Ethics Committee of UHS, Ministry of Health, Lao PDR (136/REC). We obtained written and verbal consent from eligible participants before beginning each interview and self-administration. They were told that the records would be anonymous and that they could withdraw at any moment without giving a reason.

### Role of the funding source

The funders had no role in the study design, data collection, and data analysis, data interpretation, or writing of the manuscript.

## Results

Between March and September 2021, 212 stakeholders out of 350 responded to the survey questionnaire. These included 91 participants from the MoH, 51 participants from international organisations, 43 from hospitals, 15 from education institutes, 6 from research institutes, and 6 from embassies. Responses to the survey were received within one week to up to 20 weeks after distribution of the questionnaire. Of the 55 key informants invited to participate, only 38 responded. They included 9 participants from the Lao MoH, 15 from different international organisations, 5 from hospitals at the central level, 4 from education institutions, and 5 from research institutions. The remaining 17 invited key informants (7 from the MoH, 4 from international organizations, 3 from the provincial level, 1 from the National Assembly, 1 from the Ministry of Finance, 1 from the Australian Embassy) did not participate because they were not available to be interviewed during the study period. Table 3 provides a summary of participants involved in this study.

**Table 3:** Number of respondents and key informants.

Organisation	Respondents (n = 212)	Key informants (n = 38)
Ministry of Health	91	9
International organisations	51	15
Hospitals	43	5
Education institute	15	4
Research institute	6	5
Embassy	6	0

We organised findings from both the survey and interviews, under three key overarching categories: (i) health policy decision making process in Lao PDR, (ii) the use of HTA outputs and other research evidence in the decision-making process and current gaps, and (iii) Key topic areas and stakeholders for HTA and evidence utilisation.

### Health policy decision making process in Lao PDR

#### The current process of decision-making

Health policy in Lao PDR is based on its National Strategic Plans, utilising government budgets as well as external funding sources. Generally, the MoH is accountable for the overall performance of the health sector. To begin with, each relevant sector develops an initial plan reflecting their current needs and priorities. Subsequently, proposals with detailed activities are prepared based on those plans and submitted for requesting budget to the MoH. Policy proposals, which were often mentioned, were those related to the development of treatment guidelines, Maternal and Child Health (MCH), health insurance packages.

Many interviewees indicated that most of the policy decisions, at health facilities, research institutes, and UHS as well as at the MoH level, were primarily informed through consultative meetings.

*‘I have been involved in hepatitis B treatment guideline development and it has still been in the process of decision making with several consultation meetings of focal point and external experts.’*

[Participant No. 2]

Before the MoH reaches a decision on whether to introduce a new vaccine or other health technologies, a series of consultation meetings are convened. These involve a multi-disciplinary technical working group. One example highlighted was the Lao National Immunisation Technical Advisory Group (NITAG) which consists of experts from the fields of paediatrics, obstetrics, epidemiology, laboratory sciences, health education, public health, infectious diseases, and health economics, among others recruited and appointed by the MoH. The NITAG has been dedicated to formulating recommendations to inform the national government's vaccination-related decisions.

*‘I was involved in the Rotavirus vaccine decision process. The NITAG tried to find more information regarding the cost and quality in addition to cost effectiveness, how much reimbursement would be appropriate for each of the 10 doses. All relevant information on strengths, weaknesses, and challenges were summarised and submitted to the MOH for consideration and the minister of MoH made the right final decision not to include in the routine national immunisation.’*

[Participant No. 5]

Based on a case study from the NITAG, the decision-making process involves regular meetings, needs assessment, priority identification, proposal development, and is based on the experience of staff. The NITAG members and their Committee Chair convene meetings at least three times a year to review vaccination-related matters. However, additional discussions can also be held in response to requests from the Expanded Program on Immunisation (EPI) or the Mother and Child Health Centre (MCHC). In the policy formulation process, the NITAG plays a pivotal role in providing evidence-based insights. It scrutinises the disease burden, costs, though not cost-effectiveness in practice, and takes other considerations into account.

**Fig. 2:**
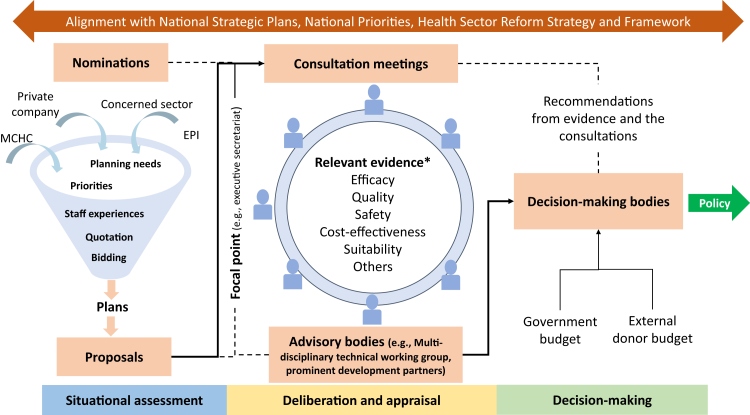
Overview of the current decision-making process in Lao PDR derived from a case study of the Lao NITAG. MCHC, Mother and Child Health Centre; EPI, Expanded Programme on Immunisation. ∗List of evidence/aspects mentioned albeit unclear how these were incorporated.

The process of consultation meetings starts with the submission of plans by MCHC to the Lao NITAG. Subsequently, the NITAG sets the meeting agenda and discussion slides which are prepared through the NITAG Executive Secretariat. The meetings normally delve into the question of whether a new vaccine should be introduced by a particular company, as well as carefully assessing its suitability for implementation in Lao PDR. Central to this deliberation process, relevant evidence was predominantly drawn from the latest recommendations provided by the WHO, along with other technical publications from various countries. It is noteworthy that in addition to the consultation meetings among the NITAG members, there was also a concerted effort to link and coordinate with other stakeholders and prominent development partners such as WHO and UNICEF, illustrating an inclusive approach for decision-making. This process, which has been developed from the inputs received in the study, is shown in Fig. 2.

#### Existing gaps in evidence use in the process

The survey findings pointed out the lack of consideration of economic aspects or value-for-money within healthcare resource allocation. Regardless, most of the respondents (90%), as presented in Table 4, identified resource allocation by the government being based on health outcome metrics such as mortality rates, nutritional well-being, severity of illness, and overall quality of life.

**Table 4:** Considerations for resource allocation for health by the government in Lao context in the past.

Items	Number	Percentage (%)
Impact on health outcomes	190	90%
Expert opinion	86	41%
Advocacy groups	101	48%
Donor priorities	88	42%
Historical basis	82	39%
Other	9	4%

N = 212.

Results from the survey generally suggested that the current health policy decision-making process in Lao PDR was perceived to be satisfactory in that, policy decisions were made in alignment with the National Strategy and Plan, considering their impact on the country. However, there were areas identified for potential improvement. Notably, there is limited information regarding the integration of health-related evidence into the decision-making processes in Lao PDR.

*‘Before we submitted the surgery equipment requirement proposal to the MoH, the consultation meeting was organised and attended by technical staff to share their opinion and experience of surgery equipment used, because we do not have research evidence to prove that it is good quality and or appropriate cost effectiveness.’*

[Participant No. 28]

Accordingly, it was also found during the interviews that decisions made were relatively less driven by the use of existing evidence/information as compared to external influence exerted by development partners or funders. Nevertheless, recently, there has been a growing interest in requiring more evidence for the process.

*‘I think decision making should be based on health evidence, not on opinion basis. Recently, the evidence base is likely increasingly required in the decision process’*

[Participant No. 15]

Policy decisions were reported as being guided by external influence. Since there is no specific research unit providing HTA output in Lao PDR, stakeholders, at health facilities, institutes, and UHS or at the MoH level, relied on information and promotions provided by drug companies due to limited local source of evidence available.

*‘I think it is not only medicines but other kinds of medical equipment that have never been assessed for their quality, efficacy and cost effectiveness by using HTA before making decision, only the information from companies and cost of products can be the best option to help in decision making, because it is not easy and not our role to do that, and there is no specific research unit to provide evidence base to us, therefore the evidence base was not applied in the decision process.’*

[Participant No. 11]

#### Recommendations for improvement of the process

Systematic approaches were suggested to be developed as an essential step towards using health evidence to inform the policy decision process. This should include a policy of evidence use development, setting criteria and agendas for HTA practice and the use of its outputs, in addition to defining the needs for evidence and mapping its demand and supply.

*‘I think decision making should be based on health evidence, not opinion-based, thereby increasing number of Lao Ministry of Health and other staff within hospitals and elsewhere being able to discuss these issues with confidence and with an evidence base is very good and important.’*

[Participant No. 15]

*In my opinion, a systematic approach with a policy of evidence use is urgently needed to apply for all health setting levels to increase the number of health evidence users in Lao [PDR]. However, defining the needs of evidence with priority setting is strongly recommended.*

[Participant No. 32]

As highlighted from the interviews, it is important that the benefits of a systematic approach are outlined and demonstrated to the MoH, including engaging with them to validate and disseminate the study outputs. This would help promote better understanding among the policymakers in terms of the rationales for having evidence embedded in the decision-making process. Given limited knowledge and expertise in HTA, tailored capacity building activities should also be in place to promote more buy-in and increase the evidence use literacy of stakeholders. For example, these may include HTA technical trainings for HTA practitioners or researchers, how-to-use evidence workshops for decision makers.

*‘Research team should be trained in this field and the research for which will be underlying what you had planned to do, so it needs more Lao colleagues who are trained at HITAP to work more cost effectively. I think HTA of the priority list of research for Lao fields could be really useful for helping to guide Lao [PDR] research right.’*

[Participant No. 38]

Although support from international partners can be useful to build local capacity of health research and HTA, it is equally important to ensure policy relevance and build an ecosystem which allows evidence to be translated into decisions.

*‘CHAI and HITAP can find a way to support you and help you to do this we would really be excited to try. I think we can do it the other way, similar to who we can provide technical assistance and help you such as CHAI. […] Where I see our support being helpful what you do after you have information available, how do we connect the result of these studies or these. These analyses connect that to policymakers, make sure it gets integrated in decision making and I think that’s where our teams are already really interested in making good evidence-based decisions, and we feel like we would have a great opportunity to partner more on that front on the side.’*

[Participant No. 30]

### The use of HTA output and other research evidence in the decision-making process and its current gaps

#### Importance of HTA and other health research evidence

HTA was acknowledged for its value in terms of informing allocating resources for health. Findings from the survey (see Table 5) revealed a consensus regarding the potential benefits of HTA in facilitating the efficient allocation of healthcare resources (71%) and enhancing healthcare quality (66%). Furthermore, respondents indicated that HTA holds promise for enhancing transparency and influencing government budgetary priorities (53% each). Interestingly, HTA was seen to have less impact towards promoting equity as compared to other elements (49%).

**Table 5:** Application of HTA for policy making in health.

Items	Strongly agree (SA) = 5	Agree (A) = 4	Moderately agree (MA) = 3	Disagree (D) = 2	Strongly disagree (SD) = 1;	Not applicable (N/A) = 0
Efficient allocation of health resources	71%	23%	5%	0%	1%	0%
Transparency in decision making	53%	39%	6%	1%	0%	0%
Impact on government budget	53%	33%	10%	3%	1%	0%
Equity	49%	34%	14%	2%	2%	0%
Financial protection	50%	37%	7%	5%	1%	0%
Improving quality of healthcare	66%	24%	9%	0%	0%	0%

N = 212.

These data were further corroborated by insights obtained from the interviews, thus reinforcing the consistency of the findings. The qualitative data underscores the significance of HTA outcomes and other empirical health research evidence within the scarce-resource context. Although HTA is a relatively novel endeavour in the country, interviewees voiced potential advantages of HTA, emphasising its pivotal role in informing policymakers for better decision-making.

*‘HTA output is very useful for policy makers to make sure their decision making is adequate, since healthcare resources are limited, and donors withdraw gradually these days.’*

[Participant No. 34]

It was observed that only a minority of participants were able to cite some examples of HTA outputs or their applications, including considerations of cost-effectiveness, cost-benefit analyses, product and healthcare quality assessments, efficiency and transparency evaluations, clinical practice guidelines, and implications for government fiscal allocations.

*‘[…] I think we need evidence of cost effectiveness, quality, safety of medicines, and common diseases in addition to considering the treatment level of the health setting, and the information from treatment guidelines, before making a decision to include some kind of medicines into the national list of essential medicines in the country. Most importantly, we need to review the treatment guidelines, because the treatment guidelines specify different kinds of medicines and usage, so we can identify the essential medicines from there, otherwise we will have useless medicine for the treatment. However, the guideline needs to be assessed and updated as well.’*

[Participant No. 5]

However, after the meaning of HTA and related concepts were explained to the interviewees, most participants strongly recommended that all aspects of HTA output should be used to support policymakers to make adequate decisions, especially due to limited healthcare resources available in Lao PDR.

*‘It could have been beneficial in leading the discussion within the MoH about research cost for Lao and Ministry of Health to have only cheaper and give direction about what's the difference research what is within Lao with national and international collaborations what should they be working on fill gaps in to inform future HTA.’*

[Participant No. 30]

In addition to supporting decision-making at the national scale, HTA can be useful for resource management in an organisational level.

*‘In my opinion, a pilot-test study in one hospital is essential to assess the cost effectiveness and possibility for policy development and implementation of the hospital authority with self-financing. HTA output of the cost effectiveness and costing for the possibility and fairness will help to indicate a good model of the hospital authority with self-financing, before scaling up to other health settings.’*

[Participant No. 28)]

Several participants also highlighted the prospective advantages of research evidence on epidemiology (prevalence, incidence, and surveillance of diseases), as well as health outcomes (mortality rates and nutritional status). They articulated that policymakers could benefit from substantial insights from such empirical data, thereby legitimising their evidence-based decisions.

*‘I think clearly about the burden of disease, the epidemiology and the impact of their time on death and disability should come from other research evidence.’*

[Participant No. 5]

Additionally, a subset of participants underscored the potential utility of evidence from routine health research. It could potentially serve as an evidential bedrock for interventions or as essential components within the HTA framework, thereby influencing policy formulation.

*‘We don't really know enough about the burden of disease for hepatitis right now, so before we could do HTA actually we have to do more formative research just around the burden of disease and understanding what we are working on. We don't know enough about how many people have hepatitis. if it's only one person in the whole country that has hepatitis maybe the $100 is not a good use of money but if it's a high risk of spreading or you know we have populations who are at risk of cancer and death because of hepatitis maybe then it's more that the benefit is higher than the cost.’*

[Participant No. 36]

#### Demand for evidence

Health professionals and several policymakers from different sectors, especially departments under the MoH were identified as key stakeholders who would use and benefit from HTA outputs. Based on the survey results, most of the respondents (46%) agreed that departments under the MoH are, or would be, the main users of HTA and other health research evidence. This was followed by hospitals and international organisations which were suggested by 25% and 17% of the respondents, respectively. See Table 6 for a summary of the results.

**Table 6:** Potential users of HTA outputs and other health research evidence.

Organisation type	Number	Percentage
Ministry of Health (including specific departments)	97	46%
Hospitals	52	25%
Research institutes and education	9	4%
International organisations	35	17%
Other centres	11	5%
Government level and National Assembly	8	4%

N = 212.

In addition to international development partners, a few ministries of the Lao government, including Ministry of Education and Sport (MoES), Ministry of Agriculture (MoA), Ministry of Finance (MoF), were also relevant as potential users of HTA outputs or other research evidence. A compiled list of HTA users, drawing from the survey and the interviews is presented in Table 7.

*‘I think that the key audience if you like the audience doing HTA in my view would be the senior policymakers in the Ministry of Health and Ministry of Finance. The primary users will be different decisionmakers in the ministry of health and people who set policy, for example, the national health insurance should be used for this which is related to the ministry of health.’*

[Participant No. 19]

**Table 7:** Summary of some relevant stakeholders of HTA outputs and other health research evidence.

Category	Sector	Organisation/person	Type of information/evidence (needed/provided)
Demand	Governmental	National/provincial Assembly	Health evidence relevant to discussion agenda
		Ministry of Health (MOH)	HTA, local clinical assessment, and other health evidence depending on their areas of mission, for example: - Vaccine-related evidence for the National Immunisation Technical Advisory Group (NITAG) - Infectious disease related evidence for Lao One Health University Network (LAOHUN)
		Technical Working Group or Commission Committee	
		Ministry of Education and Sport (MOES)	Health/health system or other research evidence that are relevant to them
		Ministry of Agriculture (MOA)	Health/health system or other research evidence that are relevant to ONE health and their mandates (e.g., food products or food security which may be linked to malnutrition)
	Academic	Hospitals and public educational institutions For example, University of Health Sciences (UHS), School of Public Health	Clinical or health system research evidence, depending on their focus.
		Independent health research institutes	
	Private	Pharmaceutical companies, Drug stores, Private clinics	HTA, clinical effectiveness, local clinical practice guidelines (CPGs)
	International organisation and NGO	World Health Organization (WHO), IRD, US-CDC, UNICEF, UNFPA, LOMWRU, KOICA, KOFIH, COPE, Gavi, Institut Pasteur du Laos, Centre d'Infectiologie Christophe Mérieux of Laos etc.	HTA, clinical effectiveness, and health system research evidence
	Other	Lay people	Clinical evidence of health technologies/interventions to increase health literacy and ensure service fairness
Supply	Governmental	Ministry of Health (MOH)	
		Food and Drug Department	Medicine and Medical products registration/information, food Safety Knowledge
		National Health Insurance Bureau (NHIB)	Health economics
		Departments of Hygiene and Health Promotion	Public Health, Health promotion/prevention, non-communicable diseases (NCDs)
		Department of Healthcare and Rehabilitation (DHR)	Public health, health system, healthcare service quality
		Public hospitals	Clinical research
	Academic	University of Health Science (UHS)	Public health, epidemiology, health economic, social science research
		Lao Tropical and Public Health Institute (TPHI)	Public health and epidemiology
	International organisations and NGO	Lao Oxford Mahosot Wellcome Trust Research Unit (LOMWRU)	Clinical research and epidemiology
		WHO	Public health, clinical guidance, and epidemiology
		Clinton Health Access Initiative (CHAI)	Public health, epidemiology, health economic assessment
		Institut de Recherche pour le Développement (IRD)	Public health and epidemiology
	Private	Indo-China research company	General research

**Abbreviations**; US-CDC, United states' Centers for Disease Control and Prevention; UNICEF, United Nations International Children's Emergency Fund; UNFPA, United Nations Population Fund; KOIKA, Korea International Cooperation Agency; KOFIH, Korea Foundation for International Healthcare; COPE, Cooperative Orthotic and Prosthetic Enterprise; GAVI, Global Alliance for Vaccine Initiative.

Some participants mentioned that collaborations between different sectors/ministries drive the demand for HTA/evidence. For example, MOA and MOH always work together for long-term solutions to nutrition issues, because the lack of food products or food security could be one of contributing factors of malnutrition in Lao PDR.

*“I think collaboration with multi-disciplinary groups would help to increase both demand and supply, especially with Ministry of Agriculture, Ministry of Education, and other concerned organisations to conduct and produce HTA and other health evidence to inform policy in Lao [PDR]*.*”*

[Participant No. 28]

Many participants mentioned that researchers and healthcare providers often demand HTA outputs and other health evidence when conducting pilots and implementing interventions. Few participants mentioned that pharmaceutical companies and laypeople would also be users for health evidence.

#### Supply of the evidence

According to the survey, most respondents (36%) indicated that the main supplier of HTA and other health research evidence in Lao PDR is academia (e.g., educational and research institutes). Almost the same proportion of the respondents (32%) also suggested that departments under the MoH can also supply the evidence, despite of being the users of it. See Table 8 for results.

**Table 8:** Potential suppliers of HTA outputs and other health research evidence.

Organisation type	Number	Percentage
Ministry of Health (including specific departments)	68	32%
Hospitals	9	4%
Research institutes and education	76	36%
International organisations	23	11%
Other centers	24	11%
Government level and National Assembly	21	10%

Note: More than one category may have been coded; percent calculated based on total number of respondents (N = 212).

During the interviews, some additional organisations/agencies were identified as suppliers of health research evidence. These included the Lao Tropical and Public Health Institute (TPHI) and the UHS. However, for HTA outputs, there were far fewer organisations named due to the lack of technical capacity and personnel in health economics. A few interviewees mentioned that collaborations and support from international partners will play a major role of driving the development of an HTA workforce in Lao PDR.

*‘It is very important to recruit really good organisations in the region that can help us to think about this. I think HITAP is the obvious first example: there are other groups from the UK called NICE international that can help and support this work.’*

[Participant No. 38]

### Key topic areas for HTA and evidence utilisation in Lao PDR

#### Key public health issues

Among many different public health areas mentioned, Maternal and Child Health (MCH) and nutrition were found to be the most important public health issues in Lao PDR (76.4% and 76.5% respectively). These areas garnered such prominence due to the high levels of maternal and child mortality, together with the malnutrition crisis within the country. Low coverage of immunisation programs, antenatal care (ANC) services, and lack of skilled birth attendants (SBA) during childbirth have collectively contributed to the rates of maternal and child mortality. There is also a high proportion of stunting, wasting and underweight population in the country. As shown in Table 9, the health system and policy landscape was also listed as a priority area for many survey respondents. Due to the COVID-19 outbreak, communicable diseases also featured. It is noteworthy that traditional medicine was also highlighted as another priority area, albeit ranked lower than other areas. Details of topics identified are presented in Table 10.

**Table 9:** Health priorities for Lao PDR.

Health priorities	Strongly agree (SA) = 5	Agree (A) = 4	Moderately agree (MA) = 3	Disagree (D) = 2	Strongly disagree (SD) = 1;	Not applicable (N/A) = 0
Mother and child health (MCH)	76%	19%	5%	0%	0%	0%
Nutrition	76%	20%	3%	1%	0%	0%
Non-communicable diseases (NCD)	42%	44%	13%	1%	0%	0%
Communicable Diseases (CDC)	46%	36%	17%	1%	0%	0%
Mental health	38%	36%	24%	3%	0%	0%
Drugs, alcohol, tobacco	30%	35%	29%	6%	0%	0%
Road traffic accident (RTA)	33%	34%	26%	6%	1%	0%
Traditional medicine	22%	36%	30%	8%	4%	0%
Medical education	46%	32%	18%	3%	1%	0%
Health system & policy	62%	28%	9%	1%	0%	0%

N = 212.

**Table 10:** Top-10 Priority areas of health in Lao PDR.

Priority area	Specific issue	Reason for priority
Maternal and Child Health (MCH)	High maternal and Child mortality	The mortality rate remains high due to low coverage of ANC/SBA
Nutrition	Malnutrition/stunting population	High proportion of population with stunting and underweight conditions
Health system and Policy	Quality of care services and accessibility to clean/safe water	Low quality and accessibility, contributing poor quality of life
Communicable Diseases (CDC)	COVID-19 and pandemic response	High socio-political-economic impacts demand effective response mechanism for the outbreak
Non-Communicable Diseases (NCD)	Hypertension, diabetes, cardiovascular disease	Increased incidence and prevalence of NCD cases
Medical education	Technical capacity of medical personnel and health literacy of the population	Upskilling workers to ensure the quality-of-care services and increasing health literacy to promote service awareness and hence accessibility
Drugs, alcohol, tobacco	Drug abuse	Increasing drug addiction with long-term impact on socio-economic aspects
Mental health	Mental health disorders	Low mental health awareness and poor mental health during COVID-19 outbreak
Road Traffic Accidents (RTA)	RTA with alcohol consumption	High rate of accidents in association with alcohol consumption
Ageing population	Quality of life among elderly	Neglected issue of elderly care and no specific department in charge of this concern

Note: the relative order of priority was ranked from high at the top to low at the bottom.

#### Policy priorities to be addressed by HTA in the future

In addition to identifying key public health priorities in Lao PDR, the survey also captured different types of policy for which HTA will be needed to inform decisions. Policies relevant to registration of health technologies, as well as service delivery for health, were indicated as the most urgent areas for HTA in the future. This was followed by the reform of provider payments, clinical practice guidelines, and the design of the basic health benefit package, including reimbursement, respectively. Within policies relevant to registration of health technologies, most respondents agreed that management of essential medicines should be assessed by HTA. Vaccines and medical devices were also emphasised as part of the top three HTA priorities, in addition to essential medicines, which can benefit from HTA practice.

While HTA has not been formally embedded in decision-making, qualitative data from the survey revealed a future focus of HTA assessment on clinical guidelines, benefits package design, health service delivery, and health service payment reform. Some participants highlighted that HTA will substantially contribute to the development of the health benefit package in Lao PDR. As in the case of the National Health Insurance Bureau (NHIB) within the MoH, relevant technical support has currently been provided by external stakeholders such as international development partners and freelance researchers.

*‘I think that the design of the basic package of health benefits is also important and urgently needs to be assessed by the HTA process, which aims at working towards UHC in the country. Assessments of costing, cost benefit, cost-effectiveness, affordability, satisfaction will help formulate an appropriate health insurance package.’*

[Participant No. 13]

Most participants mentioned a pressing need for assessing registration of health technologies. This is because such assessment would be a systematic way of documenting extant resources, medical equipment, and essential medicines. Several respondents highlighted that registration assessment offers a mechanism for scrutinising the operational efficacy of medical technologies, facilitating prioritisation of acquiring new medical technologies or replacing existing ones, thus enabling well-informed decisions.

Some participants highlighted the significance of reimbursement assessment, citing its pivotal role in determining appropriate costs for reimbursement, which serves to establish nationwide standardisation and equitable treatment of consumers. Moreover, they emphasised that such assessment could enhance financial efficiency through effective utilisation of available funds.

*‘In my opinion, reimbursement of the same medical service has still been at a different price recently. I think assessment of how effective reimbursement can help to increase the standard of the medical service means that the price would be more adequate at all levels of health setting.’*

[Participant No. 9]

Clinical guidelines are imperative to facilitate the seamless execution of HTA, given the dynamic evolution of technologies and diseases. Many participants articulated the urgency of updating clinical guidelines to align with national standards pertaining to patient safety, optimal treatment protocols, and health-related outcomes.

*‘I think infectious [disease] guidelines should be assessed, because as infectious diseases change over time, those guidelines are only really technically valid for now, or when the only evidence base available is the guidelines in the UK, the British national formulary that changes every six months.’*

[Participant No. 38]

However, a subset of respondents also pointed out that existing diagnostic and treatment practices are insufficient and diverge across varying healthcare tiers due to the absence of comprehensive clinical guidelines at the national level. Therefore, comprehensive assessment and enhancement of prevailing clinical guidelines should be in place, ensuring their applicability and alignment with the current healthcare landscape of Lao PDR.

*‘At the lower level of healthcare service, health providers still have problems with diagnosis and treatment due to lack of standard and updated guidelines nationwide. Only symptoms were reported without diagnostic and appropriate treatment. Therefore, clinical guidelines must be the same standard and updated for all levels.’*

[Participant No. 13]

## Discussion

This study is the first assessment of HTA and current as well as potential use of evidence-informed policy for health in Lao PDR. The study shows that currently, decision-making for health is centered on the national planning process, whereby programmes submit requests for planned activities. Decisions tend to be based on consultative meetings with multiple stakeholders, as observed in the case of the NITAG. Typically, decisions tend to be “opinion-based” and not “evidence-based” or “evidence-informed”, although there is an increasing appetite for using evidence to inform decisions in health. Respondents appear to be receptive to the use of HTA in the Lao context even as economic considerations are yet to be taken into account on a routine basis. Users of evidence on health, and potentially HTA, are the MoH, hospitals and international organisations. Suppliers of evidence include research institutes and UHS, although capacity to conduct HTA is low currently. In terms of topics, MCH and nutrition were identified as priority topics. Potential applications of HTA identified were development of clinical guidelines and health benefits package design among others.

There is a recognition for the importance of HTA in Lao PDR by stakeholders, yet institutional capacity for HTA remains limited. Previous studies have highlighted the importance of better communication between researchers and policymakers and this is an area that could be strengthened further.^26^^,^^27^ A regional study on the factors conducive to the development of HTA in Asia found that during the early stage of HTA development, commitment from HTA teams to develop their skills, even with part-time staff, to develop their processes can be beneficial.^28^ In many countries in Asia, having a mandate for UHC provided the policy imperative for implementing HTA to ensure sustainability of their health system.^28^, ^29^, ^30^

Building capacity of the producers of HTA, through short, medium and long-term strategies has emerged as one of the key issues. On-the-job training is an effective vehicle for learning, providing researchers with exposure to real-world demands of the discipline. This has been found to be an effective strategy to be able to meet current demand for evidence and to strengthen capacity of researchers.^31^ Having opportunities for professional growth, in terms of career opportunities in the field of HTA (including full-time positions) as well as for further studies may be a means of retaining staff who have been trained. Additionally, longer term strategies, such as introducing HTA modules in coursework for pharmacists and medical students can create a pool of future HTA researchers. In the context of Lao PDR, it may be helpful to have more training opportunities with Thailand due to similarity in the language.

Decision-making remains reliant on expert-based approaches and in the short term, the drive to establish HTA is limited. Further, the process of decision-making may not be entirely explicit, as has been observed in other countries.^32^ Raising awareness of users of HTA will be critical and involving stakeholders in the development of the HTA process can be beneficial, as was done in Bhutan.^33^ Conducting HTA studies to demonstrate their value and use for policymaking can also be a means of garnering support, as has been done in several countries. Additionally, engaging in strategic communication with policymakers using instruments such as policy briefs can be beneficial.^11^^,^^34^ The study results can be used as a starting point to identify priority topics, and additional considerations such as local political, economic, environmental, and epidemiological factors can be taken into account. Countries such as India have conducted workshops on developing policy briefs for communicating with policymakers. This may be one of the ways of engaging with policymakers in Lao PDR, to increase their engagement with evidence generated through HTA. Additionally, trainings for users of HTA such as the NHIB, MoH will also be important in increasing awareness of HTA and its potential for policy.

The process for use of evidence in Lao PDR, as shown in Fig. 2 can be linked to the public policy process, as outlined in the stages heuristics framework, which considers four stages in the public policy process: agenda setting, formulation, implementation, and evaluation.^35^ HTA can be linked to this process more generally, for example, through nomination, under agenda setting; stakeholder consultations and assessment under formulation; decision making; and implementation and evaluation. However, we would like to note that it is not always the case that an HTA agency is responsible for implementation and evaluation and it is typically only involved in upward rather than downward policy development. Further, it may be noted that the policy cycle also does not explicitly incorporate researcher participation.

Data constraints are evident and are a common challenge in LMICs and was also identified as a challenge in ASEAN countries for conducting HTA.^32^ However, this need not be a limitation that cannot be overcome and at the initial stage, data could be borrowed from other settings.^28^ Further, it is possible that once data are used for policy, their quality will improve. Building the infrastructure for better data requires long term investment; for example, countries such as Thailand and India developed a costing menu to increase availability of local data and reduce the need for primary data collection for each study.^36^^,^^37^

Importantly, financial resources will need to be made available to develop HTA in the country and the cost for establishing such institutions can vary from one country to another.^28^^,^^34^ HITAP in Thailand received a seed grant of USD 1 million for the first three years and has since received funding from users to conduct assessments.^38^ Given the limited financial resources for health, particularly in the aftermath of the COVID-19 pandemic, where there has been a sharp economic downturn, there is a dependence on donor funding. While there is a need to mobilise resources domestically, or use existing resources economically, this may not transpire in the short term. There is an urgent need for international donors to support policy-related processes, instead of programme-oriented support. Potential options are to consider bilateral donor agencies or those donors focused on building research capacity.

The study highlighted the importance of collaborations with other countries. Language is potentially a key consideration. Increasing partnerships in the region can increase the longer-term sustainability of collaborations. In addition, there are opportunities for Lao PDR to participate in regional networks such as HTAsiaLink and engage with partners on initiatives such as harmonisation of HTA through ASEAN, which can potentially reduce duplication of efforts.^12^ Such collaborations can also utilise the capacity for HTA to support other relevant initiatives such as harmonisation of vaccination schedules and pooled procurement.

Given the early stage of HTA in the country, this study points to the potential and importance of establishing HTA. The establishment of UHEP was to further the use of HTA in the country, with the support of the Wellcome Trust and the UK Department of Health and Social Care. UHEP's set-up is guided by this situational analysis which offered insights on the HTA ecosystem in the country, on the use of evidence for health policy and key policy processes and capacities that support evidence uptake. The situational assessment also provides an indication of the immediate health needs and priority issue areas for Lao PDR allowing a better utilisation of available resources.

Since the establishment of UHEP, there have been multiple activities related to HTA in Lao PDR: an online stakeholder consultation on HTA to launch UHEP, a stakeholder consultation and topic prioritisation workshop in September 2022^39^; an HTA workshop in July 2023, in collaboration with partners. Researchers from Lao PDR have been supported to pursue doctoral programmes in Thailand on HTA and related topics as well as internship programmes. Further, staff from Lao PDR have joined trainings on vaccinology and on conducting economic evaluations.^40^ An economic evaluation of typhoid vaccine, identified as a priority topic as it had been proposed through Gavi, has been completed^41^ and in the current phase, an economic evaluation of renal replacement therapy in Lao PDR is underway. These studies are using an on-the-job training approach, in collaboration with partners across countries.

It may be noted that there are different types of decision making for health. Some of the issues found above may be generalisable to other areas of decision making. In HTA, there is an emphasis on taking a multidisciplinary approach and to be participatory, for example. However, this may not apply to other areas of health decision-making. For example, if considering the role of a health regulatory agency, it can focus on clinical aspects and does not need to incorporate economic or social issues (though, it may take ethical issues into account). Further, such agencies do not need to be participatory and can focus on regulators and manufacturers.

This study has some limitations. It was conducted during the COVID-19 pandemic and as such, fielding the survey and conducting in-person interviews was not feasible. This affected the sample data collected. Due to resource and time constraints, formal assessments of the questionnaire were not conducted. Furthermore, HTA is a relatively new concept in Lao PDR, hence the understanding of what can be achieved by using HTA may be limited, based on the brief explanation provided to participants in the survey.

This study adds to the literature on conducting situation analyses of HTA in countries. It adapts an existing questionnaire to the country context and employs a mixed methods approach to elicit the responses. The questionnaire took a broader perspective on evidence use for policy to understand the use of evidence for policy and integrated elements of HTA into the process. This approach may be helpful for other countries that are taking steps towards institutionalising HTA and are seeking to better understand opportunities.

## Contributors

YT and MM conceptualised the study and provided supervision. SP and AA developed the study design, with inputs from YT, MM, EA and SD. SP, SV, SS conducted the study and the analysis. SP and MM directly accessed and verified underlying data reported in the manuscript. MS, AA, SP, SD wrote first drafts of sections of the manuscript. EA, YT, MM reviewed and edited the manuscript. SP, SV, SS had access to and verified the data collected from the survey and interviews; MS and SD had access to the raw data for the survey. SD had final responsibility for the decision to submit for publication.

## Data sharing statement

Data is available upon request.

## Declaration of interests

The authors do not have any conflicts of interest to declare.
