# Supplementary material for: Situation analysis of evidence-informed health decision-making in Lao PDR: the case of health technology assessment
Source: Lancet Reg Health West Pac. 2025 Apr 9;57:101534. doi: 10.1016/j.lanwpc.2025.101534 (PMC12008126; doi:10.1016/j.lanwpc.2025.101534)
Supplement: Appendix 1 [file mmc1.pdf]

Appendix

Appendix 1: Survey questionnaire on HTA and other health evidence to inform policy

The University of Health Sciences, Ministry of Health, Lao PDR, is leading to establish the Unit of Health Evidence and Policy (UHEP). We would like to invite you to take this survey to hear your thoughts on Health Technology Assessment (HTA) and health evidence to inform policy in Lao PDR. The results of this survey will be anonymous and will be very useful for further implementation of the UHEP in Lao PDR.

Objective of the Survey

The survey forms an important component in the establishment of UHEP in Lao PDR, as a first step towards understanding the landscape and processes of current evidence (including health technology assessment) in informing health decisions, as well as the opportunities and challenges in doing so in the future. Your responses will contribute towards progress in evidence-informed health policy in the country and be very useful for further implementation of the UHEP in Lao PDR.

Structure of the Survey and Instructions to Respondents

We have adapted this questionnaire from the "Situation Analysis of HTA Introduction at National Level" developed by HITAP and NICE International. “HTA is a multidisciplinary process that uses explicit methods to determine the value of a health technology at different points in its lifecycle. The purpose is to inform decision-making in order to promote an equitable, efficient, and high-quality health system”.

This survey is divided into the following sections: ການສໍາຫຼວດນີ້ແບ່ງອອກເປັນພາກຕ່າງໆດັ່ງຕໍ່ໄປນີ້:

Section I: Need for HTA or other relevant evidence in your context

Section II: Demand for HTA and other relevant evidence in your context

Section III: Supply of HTA and other relevant evidence in your context

Section IV: Opportunities for your organization with HTA and other health evidence

Each section should take approximately 5 minutes to answer, and the survey must be completed in one sitting. The results of this survey will be anonymous.

We suggest that you may think of one context in Lao PDR - national, municipal/provincial, or other while answering each part of this survey.

Thank you for participating in our survey. If you have any questions, please write to us at

Name: Dr. Sysavanh Phomachanh

Position: Vice-Director of the Institute of Research and Education Development (IRED)

Organisation: The University of Health Sciences. Email: sysavanhp@gamail.com

Section I: Need for HTA and other relevant evidence in your context

1. In your opinion, how are resources for healthcare allocated by the government in Lao context in the past? (select all that apply) ❑ Impact on health outcomes ❑ Expert opinion ❑ Advocacy groups ❑ Donor priorities ❑ Historical basis ❑ Others, please specify..

The following questions will seek your opinion on **the role of Health Technology Assessment (HTA)** which is defined as "a **multidisciplinary process** that uses explicit methods to determine the value of a health technology at different points in its lifecycle. The purpose is to inform decision-making in order to promote an equitable, efficient, and high-quality health system." For example,

This section, question number 2 to 5, please indicate how you agree or disagree with each statement by circling the number corresponding to your response. Use the following scale to indicate "Strong Agree that the information is very important (SA)=5; A=Agree that the information is important (A)=4; Moderate Agree that the information is important (MA)=3; D= Disagree that the information is important (D)=2; SD=Strong Disagree that the information is very important (SD)=1; Not applicable (N/A)=0

2. HTA helps address several aspects of policy making in health. Please rate each of the attributes listed below in terms of their importance to you.

**Statement**	**SA**	**A**	**MA**	**D**	**SD**	**N/A**
Efficient allocation of health resources	5	4	3	2	1	0
Transparency in decision making	5	4	3	2	1	0
Impact on government budget	5	4	3	2	1	0
Equity	5	4	3	2	1	0
Financial protection	5	4	3	2	1	0
Improving quality of healthcare	5	4	3	2	1	0
Other, please specify	5	4	3	2	1	0
Other, please specify	5	4	3	2	1	0
Other, please specify	5	4	3	2	1	0

3. HTA can be used in different health policy areas to improve the evidence base for decision making. In your opinion, please rate each of the following policy areas based on the importance of HTA evidence towards each.

**Statement**	**SA**	**A**	**MA**	**DA**	**SD**	**N/A**
Registration of individual health technologies	5	4	3	2	1	0
Reimbursement of individual health technologies	5	4	3	2	1	0
Clinical guidelines or disease management pathways development	5	4	3	2	1	0

Design of basic package of health benefits	5	4	3	2	1	0
Service delivery for Health	5	4	3	2	1	0
Reform of provider payment systems	5	4	3	2	1	0
Other, please specify	5	4	3	2	1	0
Other, please specify	5	4	3	2	1	0
Other, please specify	5	4	3	2	1	0

4. HTA can be used to assess different types of health technologies. In your opinion, please rate each of the following policy areas based on the importance of HTA evidence in each

**Statement**	**SA**	**A**	**MA**	**DA**	**SD**	**N/A**
Medicines	5	4	3	2	1	0
Vaccines	5	4	3	2	1	0
Medical devices	5	4	3	2	1	0
Screening programs	5	4	3	2	1	0
Referral programs	5	4	3	2	1	0
Procedures by health professionals (e.g. surgeries)	5	4	3	2	1	0
Public health programs or initiatives	5	4	3	2	1	0
Service delivery initiatives or incentives	5	4	3	2	1	0
Other, please specify	5	4	3	2	1	0
Other, please specify	5	4	3	2	1	0
Other, please specify	5	4	3	2	1	0

5. What other health priorities are important to inform policy makers? Please rate each of the areas listed below:

**Statement**	**SA**	**A**	**MA**	**DA**	**SD**	**N/A**
Mother and child health (MCH)	5	4	3	2	1	0
Nutrition	5	4	3	2	1	0
Non-communicable diseases (NCD)	5	4	3	2	1	0
Communicable Diseases (CDC)	5	4	3	2	1	0
Mental health	5	4	3	2	1	0
Drug, alcohol, tobacco	5	4	3	2	1	0
Road traffic accident (RTA)	5	4	3	2	1	0
Traditional medicine	5	4	3	2	1	0
Medical education	5	4	3	2	1	0
Health system & policy	5	4	3	2	1	0
Other, please specify	5	4	3	2	1	0
Other, please specify	5	4	3	2	1	0
Other, please specify	5	4	3	2	1	0

Section II: Demand for HTA and other relevant health evidence in your context

1. Please list ONE organization that is a potential user of HTA and/or other relevant health evidence areas in Lao context ....
2. At which level does this organization operate: ❑ National ❑ University ❑ MOH ❑ Provincial ❑ Other, please specify ....
3. Based on the organization chosen above, please rate how important the following attributes are for HTA and evidence-based priorities answered in Section 1.

Question number 3, please indicate how you agree or disagree with each statement by circling the number corresponding to your response. Use the following scale to indicate "Strong Agree that the information is very important (SA)=5; A=Agree that the information is important (A)=4; M/F=Moderate Agree that the information is important (MA)=3; D= Disagree that the information is important (D)=2; SD=Strong Disagree that the information is very important (SD)=1; Not applicable (N/A)=0

**Statement**	**SA**	**A**	**MA**	**DA**	**SD**	**N/A**
Safety	5	4	3	2	1	0
Efficacy	5	4	3	2	1	0
Cost Effectiveness	5	4	3	2	1	0
Budget Impact	5	4	3	2	1	0
Social and ethical considerations	5	4	3	2	1	0
Other, please specify	5	4	3	2	1	0
Other, please specify	5	4	3	2	1	0
Other, please specify	5	4	3	2	1	0

4. Please identify training and capacity building needs to improve HTA capacity of evidence users *(rank from highest to lowest in importance; please add suggestions for other trainings in the space provided)*:

❑Introduction and Application of HTA

0	1	2	3	4	5	6	7	8	9	10

Not important Very important

❑Topic HTA Selection Process for HTA

0	1	2	3	4	5	6	7	8	9	10

Not important Very important

❑Overview of Health Economics

0	1	2	3	4	5	6	7	8	9	10

Not important Very important

❑Institutional processes for HTA

0	1	2	3	4	5	6	7	8	9	10

Not important Very important

❑ Other, please specify . ...

0	1	2	3	4	5	6	7	8	9	10

Not important Very important

Section III: Supply of HTA and other relevant health evidence in your context

1. Please identify ONE organization that supplies or generates health evidence to support health policy decisions in your context...
2. At which level does this organization operate: ❑ National ❑ University ❑ MOH ❑ Provincial ❑ Other, please specify ....
3. Based on the organization chosen above, please rate how important the following attributes are for HTA and evidence-based priorities answered in Section 1.

This section, question number 3, please indicate how you agree or disagree with each statement by circling the number corresponding to your response. Use the following scale to indicate "Strong Agree that the information is very important (SA)=5; A=Agree that the information is important (A)=4; M/F=Moderate Agree that the information is important (MA)=3; D= Disagree that the information is important (D)=2; SD=Strong Disagree that the information is very important (SD)=1; Not applicable (N/A)=0

**Statement**	**SA**	**A**	**MA**	**DA**	**SD**	**N/A**
Safety	5	4	3	2	1	0
Efficacy	5	4	3	2	1	0
Cost Effectiveness	5	4	3	2	1	0
Budget Impact	5	4	3	2	1	0
Social and ethical considerations	5	4	3	2	1	0
Other, please specify	5	4	3	2	1	0
Other, please specify	5	4	3	2	1	0
Other, please specify	5	4	3	2	1	0

4. Availability of local data to inform country-specific decisions is a key challenge for conducting HTA and generating other relevant health evidence. Please indicate the availability of the following types of data:

NA=Not available, AL=Available with limitation, A=Available

**Statement**	**NA**	**AL**	**A**
Pharmaceutical usage and pricing			
Hospital level data (e.g. utilization rates)			
Health outcomes (e.g. mortality, QALYs) ຂໍ້			
Çosts of service delivery (for e.g. salaries of health professionals) ຂໍ			
Social and ethical considerations			
Other, please specify			

5. What type HTA infrastructure should be available in Lao context: *(rank in order of importance; please add suggestions in the space provided*)

❑Methodological guidelines for HTA

0	1	2	3	4	5	6	7	8	9	10

Not important Very important

❑Institutional processes for HTA

0	1	2	3	4	5	6	7	8	9	10

Not important Very important

❑Database of HTA studies

0	1	2	3	4	5	6	7	8	9	10

Not important Very important

❑Decision Criteria (threshold) for HTA

0	1	2	3	4	5	6	7	8	9	10

Not important Very important

❑Health management information systems (HMIS)

0	1	2	3	4	5	6	7	8	9	10

Not important Very important

❑Other, please specify.............

0	1	2	3	4	5	6	7	8	9	10

Not important Very important

6. What other types of health research evidence infrastructure should be available in Lao context: *(rank in order of importance; please add suggestions in the space provided*)

❑Methodological guidelines for other health evidence

0	1	2	3	4	5	6	7	8	9	10

Not important Very important

❑Institutional processes

0	1	2	3	4	5	6	7	8	9	10

Not important Very important

❑Database of other research studies

0	1	2	3	4	5	6	7	8	9	10

Not important Very important

❑Decision Criteria (threshold)

0	1	2	3	4	5	6	7	8	9	10

Not important Very important

❑Health management information systems (HMIS)

0	1	2	3	4	5	6	7	8	9	10

Not important Very important

❑Other, please specify.......................

0	1	2	3	4	5	6	7	8	9	10

Not important Very important

7. Please identify training needs to improve research capacity of evidence generators *(rank in order of importance; please add suggestions in the space provided)*:

❑Introduction and Application of other health research areas

0	1	2	3	4	5	6	7	8	9	10

Not important Very important

❑Research methodology

0	1	2	3	4	5	6	7	8	9	10

Not important Very important

❑Systematic Reviews

0	1	2	3	4	5	6	7	8	9	10

Not important Very important

❑Meta-analysis ການວິເຄາະຜົນການຄົ້ນຄວ້າທີ່ເຮັດຜ່ານມາແລ້ວ

0	1	2	3	4	5	6	7	8	9	10

Not important Very important

❑Measuring Health Outcomes

0	1	2	3	4	5	6	7	8	9	10

Not important Very important

❑Scientific writing

0	1	2	3	4	5	6	7	8	9	10

Not important Very important

❑Policy brief writing

0	1	2	3	4	5	6	7	8	9	10

Not important Very important

❑Other, please specify................................. ....

0	1	2	3	4	5	6	7	8	9	10

Not important Very important

Section IV: Opportunities for your organization with HTA and other relevant health evidence

1. Please indicate the type of your organization *(select one)*: ❑Government (ministry, autonomous institution, others), ❑Private sector ❑Not-for-profit non-governmental organization ❑Research institute ❑Academic institute ❑Multilateral/bilateral agency ❑Other, please specify..................
2. Please provide the location (city/town/state) of your organization: ....
3. At which level does your organization operate? ❑ National ❑ University ❑ MOH ❑ Provincial ❑ Other, please specify ....
4. Do you see your organization as a generator or user of HTA evidence? ❑Generator ❑User ❑Both ❑Other, please specify ....
5. Please provide ONE health intervention topic that can potentially be an HTA research topic...
6. Please prioritize three most important research topics to inform policy

   ……………………………………………………………………………………………
   ……………………………………………………………………………………………
   ……………………………………………………………………………………………

7. For the priority topic you provided above, please suggest at least ONE research question that you think should be examined.

   ……………………………………………………………………………………………

8. Please identify criteria for consideration: ❑high probability of impact of research, ❑capacity to conduct study, ❑feasibility of study within time frame of 1.5 years. ❑ Other, please specify ....

9. Please identify your organization's strengths in terms of using and/or generating evidence for HTA and other health research evidence
....
....

10. Please identify the constraints your organization faces in terms of using and/or generating evidence for HTA and other health research evidence

....
....

11. Do you have any additional comments?...
....

Thank you for your participation in this survey!
