# Supplementary material for: Situation analysis of evidence-informed health decision-making in Lao PDR: the case of health technology assessment
Source: Lancet Reg Health West Pac. 2025 Apr 9;57:101534. doi: 10.1016/j.lanwpc.2025.101534 (PMC12008126; doi:10.1016/j.lanwpc.2025.101534)
Supplement: Appendix 2 [file mmc2.pdf]

Appendix 2: Interview guideline for policy makers/Director/academic staff /development partners

CATEGORY	DESCRIPTION
Interviewee	Concerned people who are involved in health decision-making at the national or provincial level and those who are familiar with health evidence (relevant stakeholders)
Interviewer & Note taker	Name.……………………………and his field assistant (primary)
	Name. …………………………... and her field assistant (secondary)
Focus 3 sections:	1). Current health policy decision making process in Laos<br>2). Need for, demand for, supply of health evidence;<br>3) strength, weakness, opportunity, and threat to establish the Unit of Health Evidence and Policy (UHEP) in Lao PDR
Objective of section 1	✓ to understand the current context of health policy decisions in Laos, and identify the current approaches for development of healthcare guidelines and determining their health benefits with examples where relevant
	✓ to understand the role of evidence in informing these decisions and identify barriers that impact the generation and utilization of this evidence
Objective of section 2	✓ to explore open opinion and thoughts on the needs for, demand for, and supply of HTA and other health research evidence in Lao PDR
Objective of section 3	✓ to understand the strengths, weaknesses, opportunities, and threats (SWOT) on establishing the Unit of Health Evidence and policy (UHEP) in Lao PDR
Date of interview	
Method	✓ individual face to face and online interview as applicable<br>✓ Two interviewers for each interviewee (One person will conduct interview and another person is note taker).
Duration	About 60-90 minutes for each interview
Place	Quiet and comfortable for the interviewee (e.g. office, coffee shop, or home/online if required)

NOTE: interrupter control & facilitator	Unwelcomed accompany	NONE
	Welcomed accompany	

STEPS

1. Build Rapport
2. Introduce yourself (interviewer) to the interviewee
   - My name is……………………………….… I am a research team from ………………………
   - Here is Dr.___ who leads the interview our interview.
   - If you allow, we will use a recorder to record our conversation/discussion, because we do not want to miss any important points.

3. Give an outline of your interview

   "The University of Health Sciences, Ministry of Health, Lao PDR, is leading the establishment of the Unit of Health Evidence and Policy (UHEP). UHEP aims to encourage the use of evidence in health policy making in Laos. As a first step towards setting up this Unit, we would like to learn and ask for your suggestions/opinions and discuss the context for (including the needs of, demand for, and supply of) health evidence to inform policy making in Lao PDR. We also want to know your thoughts on the strengths, weaknesses, opportunities, and threats on how UHEP can be established in the country. These conversations are an important step towards ensuring more equitable, efficient health decisions in the country, with available resources."

   We have 3 parts of questions – 1). Current health policy decision making process in Laos 2). Need for, demand for, supply of health evidence; 3) strength, weakness, opportunity, and threat to establish the Unit of Health Evidence and Policy (UHEP) in Lao PDR. We expect the interview process will take around 60-90 mins. This interview is voluntary and anonymous, if you would like to pause or stop the interview at any time, please let us know.

   Fill the consent form if interviewee agrees, and begin the interview by asking the following questions:

Section :1 General questions on health policy in Lao context

1. Could you please share your though, how is a health policy decision in the country actually made at the moment?
   - If possible, could you provide an example of a health policy and the process of decision-making? (In case they could not think of those examples, the interviewer provides them one of these examples related to health infrastructure and human resources development, law and regulation, medical products "vaccines, medicines, medical devices", or service

delivery" (For example, before introducing drug or medicine into the health insurance scheme, what do people do? In Lao context)
- Who is the decision-maker?
- What are the factors influencing the decision made? Who influences the decision-making process?

2. What is the current approach, by who and why? (Ask them to give an example if possible)
3. Do you think people are happy or satisfied with the current approach? Why or why not?
4. Do you think this approach will continue in the future? Why or why not?
5. Are there any other approaches that you would like to propose? Why?
6. What are the key public health issues in Laos? Could you please identify two specific priority areas for health or health care in the country? Why are these issues important?
7. How would you ***understand*** evidence-informed policymaking in this context? For instance, what are some of the key factors, characteristics, or determinants you would associate with using evidence in health policy decisions?

Section 2: Opinion toward the needs for, demand for, and supply of HTA and other health research evidence in Lao PDR

2.1 The needs for HTA and other health research evidence

1. Are you aware of Health Technology Assessment (HTA)? How would you describe it?
2. Do you think health technology assessment (HTA) is a useful policy device? If yes, ***how and why?***
3. What other health research evidence can be useful to health policy decision in the country? Why?
4. Which policy area types of HTA output are urgently needed in the country?
   -registration of health technologies, why?
   -coverage or reimbursement of individual health technologies, why?
   -production of clinical guidelines or disease management pathway, why?
   -informing design of basic package of health benefits, why?
   -health service delivery design, why?
   -provider payment reform or pay for performance schemes, why?
5. Which output of HTA process is urgently needed in the country?
   -medicine, why?
   -vaccination, why?
   -medical device/diagnostic, why?
   -other interventions (e.g. surgical procedures, etc)
   -screening and referral programs, why?
   -public health programs or initiatives, why?
   -service delivery initiative or incentives, why?
6. What are the research questions that you believe can be answered by HTA for priority topic aboved?
7. If there are budget constraints, all proposed health investments cannot be supported and prioritisation is needed. Which criteria do you think could be used for prioritising the technology investments? (For example, criteria could include ethics and social justice, cost-effectiveness, disease prevalence or others). Please rank the criteria in order of relevance with the most important first.

8. What do you think are the main barriers to establishing investments in the health technologies/interventions in the Laos context?

2.2 The demand for HTA and other health research evidence

1. Do you think, who are the potential users of HTA and other health evidence in the country?
2. Do you think What organizations require health evidence usually in the country? Why?
3. Do you think who and which organization usually are interested in HTA or other health evidence? Could you please identify the individual, organisation or stakeholder interested in HTA or other health evidence? How would this information help with the decision-making process?

2.3 The supply of HTA and other health research evidence

1. Could you please identify the individuals or organizations that supply or generate evidence to support health policy decisions in the country? If possible, provide the names of organizations and contact persons. What are the roles of these organizations that can supply evidence?
2. What are the types of local data available to inform health policy decisions?
   To interviewer: Based on the interviewee's knowledge, consider providing examples to help contextualise, for instance, availability of cost information for health products and services, disease incidence/prevalence, health service utilization and any others?
3. Would you say access to data is a key challenge in pursuing HTA and other evidence informed decision-making processes? Based on the above answer, which types of data is hardest to access in the country?
4. What do you think about having an institution like UHEP in Lao PDR? Why?
5. What types of infrastructure are required to establish UHEP in Lao PDR?
6. Could you please suggest, are there any specific training or capacity building needs to initiate HTA and other evidence based health policy processes in Lao?

Section3: Concluding questions using SWOT

We will discusss with you on the strengths, weaknesses, opportunities, and threats to establishing a body like UHEP in Laos PDR?

- Strengths: …………….

-Weaknesses:……………

-Opportunities…………..

-Threats/barriers…………

Wrap up the conversation:

- ✓ Say thank you to the interviewee for participating and providing their valuable time towards this discussion.
- ✓ Give a summary of the interview and outline the most important information we have received from the interviewee.

- ✓ Ask the participants if the interpretation of the received information is correct and if they want to add anything and ask them if they have any comments or suggestions from today's conversation.
- ✓ Provide an update of how results from the interview will be disseminated or shared? Any information on stakeholder meetings to be given.
- ✓ Finding will be share throughout the first workshop with some of you who are invited to attend, which aims validation of information. Thereafter, the end of this project, public dissemination will be organized in Vientiane Capital, and publication will be done for scientific knowledge.

Close your conversation:

Thank you very much for your participation and sharing information and experiences with us-really appreciated. We may contact with you again if any missing information is required!

Do after your Interview:

- Check if the conversation has been appropriately recorded.
- Fill the blank in the following table on next page immediately after the IDI: Please make a brief note on context and any other relevant information regarding the interview before you moving onto the next one

CATEGORY	DESCRIPTION
Interviewee	Individual:
Interviewer	NAME
Date of interview	
Time length of interview	
Place of interview	
Observations of assistant	1. How cooperative were the participants?<br>2. How comfortable do you think were the participants in discussing the topics?<br>3. How knowledgeable were the participants about issues that were discussed in this interview?<br>4. Are there any specific comments about this interview?
