# Supplementary material for: Situation analysis of evidence-informed health decision-making in Lao PDR: the case of health technology assessment
Source: Lancet Reg Health West Pac. 2025 Apr 9;57:101534. doi: 10.1016/j.lanwpc.2025.101534 (PMC12008126; doi:10.1016/j.lanwpc.2025.101534)
Supplement: Appendix 3 [file mmc3.pdf]

Appendix 3
Survey responses (response to close-ended questions)

Section I: Need for HTA and other relevant evidence in your context

1. In your opinion, how are resources for healthcare allocated by the government in Lao context in the past? (select all that apply)

Considerations	Number
Impact on health outcomes	190
Expert opinion	86
Advocacy groups	101
Donor priorities	88
Historical basis	82
Other	9

2. HTA helps address several aspects of policy making in health. Please rate each of the attributes listed below in terms of their importance to you.

Attributes	Strong Agree (SA)=5	Agree (A)=4	Moderate Agree (MA)=3	Disagree (D)=2	Strongly Disagree (SD)=1;	Not applicable (N/A)=0	Total
Efficient allocation of health resources	150	49	11	0	2	0	212
Transparency in decision making	113	82	13	3	1	0	212
Impact on government budget	113	69	22	6	2	0	212
Equity	103	72	29	4	4	0	212
Financial protection	105	78	15	11	3	0	212
Improving quality of healthcare	140	51	19	1	1	0	212

3. HTA can be used in different health policy areas to improve the evidence base for decision making. In your opinion, please rate each of the following policy areas based on the importance of HTA evidence towards each.

Health policy areas	Strong Agree (SA)=5	Agree (A)=4	Moderate Agree (MA)=3	Disagree (D)=2	Strongly Disagree (SD)=1;	Not applicable (N/A)=0	Total
Registration of individual health technologies	145	50	12	1	4	0	212
Reimbursement of individual health technologies	105	74	23	3	7	0	212
Clinical guidelines or disease management pathways development	116	65	27	4	0	0	212
Design of basic package of health benefits	115	70	21	6	0	0	212
Service delivery for Health	132	54	17	7	2	0	212
Reform of provider payment systems	120	61	19	7	5	0	212

4. HTA can be used to assess different types of health technologies. In your opinion, please rate each of the following policy areas based on the importance of HTA evidence in each

Policy areas	Strong Agree (SA)=5	Agree (A)=4	Moderate Agree (MA)=3	Disagree (D)=2	Strongly Disagree (SD)=1;	Not applicable (N/A)=0	Total
Medicines	153	44	13	2	0	0	212
Vaccines	141	54	13	4	0	0	212
Medical devices	131	68	11	2	0	0	212
Screening programs	122	64	20	2	4	0	212
Referral programs	104	73	24	9	2	0	212
Procedures by health professionals (e.g. surgeries)	97	76	31	8	0	0	212
Public health programs or initiatives	117	59	29	5	2	0	212
Service delivery initiatives or incentives	104	73	28	3	4	0	212

5. What other health priorities are important to inform policy makers? Please rate each of the areas listed below:

Health priorities	Strong Agree (SA)=5	Agree (A)=4	Moderate Agree (MA)=3	Disagree (D)=2	Strongly Disagree (SD)=1;	Not applicable (N/A)=0	Total
Mother and child health (MCH)	162	40	10	0	0	0	212
Nutrition	162	42	6	2	0	0	212
Non-communicable diseases (NCD)	89	93	28	2	0	0	212
Communicable Diseases (CDC)	97	76	36	3	0	0	212
Mental health	80	76	50	6	0	0	212
Drug, alcohol, tobacco	63	74	62	13	0	0	212
Road traffic accident (RTA)	70	72	55	13	2	0	212
Traditional medicine	46	77	64	16	9	0	212
Medical education	97	67	38	7	3	0	212
Health system & policy	131	60	19	2	0	0	212

Section II: Demand for HTA and other relevant health evidence in your context

1. Please list ONE organization that is a potential user of HTA and/or other relevant health evidence areas in Lao context
Coded responses

Organisation type	Number
Ministry of Health (including specific departments)	97
Hospitals	52
Research institutes and education	9
International organisations	35
Other centers	11
Government level and National Assembly	8

N = 212

2. At which level does this organisation operate:

Level	Number
National	167
University	8
Organisational	3
Provincial	14
Hospital level	10
International	9
N/A	1
Total	212

Appendix 3
Survey responses (response to close-ended questions)

3. Based on the organization chosen above, please rate how important the following attributes are for HTA and evidence-based priorities answered in Section 1.

Attributes	Strong Agree (SA)=5	Agree (A)=4	Moderate Agree (MA)=3	Disagree (D)=2	Strongly Disagree (SD)=1;	Not applicable (N/A)=0	Total
Safety	148	57	5	0	2	0	212
Efficacy	136	64	10	0	2	0	212
Cost Effectiveness	128	65	19	0	0	0	212
Budget Impact	125	55	25	3	4	0	212
Social and ethical considerations	124	60	18	10	0	0	212

4. Please identify training and capacity building needs to improve HTA capacity of evidence users (rank from highest to lowest in importance; please add suggestions for other trainings in the space provided): (0 = Not important; 10 = Very important)

Type of training	0	1	2	3	4	5	6	7	8	9	10	Total
Introduction and Application of HTA	0	0	0	0	0	5	4	10	43	70	80	212
Topic HTA Selection Process for HTA	0	0	0	0	2	4	3	17	48	73	65	212
Overview of Health Economics	0	0	0	0	3	4	7	22	49	51	76	212
Institutional processes for HTA	0	0	0	0	0	3	7	14	45	67	76	212
Other	13	18	0	0	0	0	0	0	0	0	0	31

Section III: Supply of HTA and other relevant health evidence in your context

1. Please identify ONE organization that supplies or generates health evidence to support health policy decisions in your context.
Coded responses

Organisation type	Number
Ministry of Health (including specific departments)	68
Hospitals	9
Research institutes and education	76
International organisations	23
Other centers	24
Government level and National Assembly	21

Note: More than one category may have been coded; percent calculated based on total number of respondents (N=212)

2. At which level does this organization operate:

Level	Number
National	162
Organisational	9
Hospital level	21
Provincial	9
University	8
International	3
Total	212

3. Based on the organization chosen above, please rate how important the following attributes are for HTA and evidence-based priorities answered in Section 1.

Attributes	Strong Agree (SA)=5	Agree (A)=4	Moderate Agree (MA)=3	Disagree (D)=2	Strongly Disagree (SD)=1;	Not applicable (N/A)=0	Total
Safety	139	64	9	0	0	0	212
Efficacy	137	67	8	0	0	0	212
Cost Effectiveness	121	68	20	3	0	0	212
Budget Impact	118	60	28	3	3	0	212
Social and ethical considerations	121	63	25	3	0	0	212

4. Availability of local data to inform country-specific decisions is a key challenge for conducting HTA and generating other relevant health evidence. Please indicate the availability of the following types of data:

Type of data	NA=Not available	AL=Available with limitation	A=Available	Total
Pharmaceutical usage and pricing	27	157	28	212
Hospital level data (e.g. utilization rates)	28	151	33	212
Health outcomes (e.g. mortality, QALYs)	32	160	20	212
Costs of service delivery (for e.g. salaries of health professionals)	49	126	37	212
Social and ethical considerations	70	130	12	212

5. What type HTA infrastructure should be available in Lao context: (rank in order of importance; please add suggestions in the space provided) (0 = Not important; 10 = Very important)

Type of infrastructure	0	1	2	3	4	5	6	7	8	9	10	Total
Methodological guidelines for HTA	0	0	0	0	1	3	4	13	43	55	93	212
Institutional processes for HTA	0	0	0	1	0	3	2	19	46	79	62	212
Database of HTA studies	0	0	1	0	1	2	4	13	56	56	79	212
Decision Criteria (threshold) for HTA	0	0	1	0	0	2	6	14	58	68	63	212
Health management information systems (HMIS)	0	0	0	1	0	4	0	14	42	72	79	212
Other	0	11	0	0	0	0	0	0	0	0	0	11

6. What other types of health research evidence infrastructure should be available in Lao context: (rank in order of importance; please add suggestions in the space provided). (0 = Not important; 10 = Very important)

Type of infrastructure	0	1	2	3	4	5	6	7	8	9	10	Total
Methodological guidelines for other health evidence	0	0	0	1	0	2	2	10	32	79	86	212
Institutional processes	0	0	0	0	0	2	4	9	60	73	64	212
Database of other research studies	0	1	0	0	0	1	4	15	60	59	72	212
Decision Criteria (threshold)	0	0	0	0	0	3	5	24	46	68	66	212
Health management information systems (HMIS)	0	0	0	0	1	0	5	13	45	72	76	212
Other	0	7	0	0	0	0	0	0	0	0	0	7

Appendix 3

Survey responses (response to close-ended questions)

7. Please identify training needs to improve research capacity of evidence generators (rank in order of importance; please add suggestions in the space provided): (0 = Not important; 10 = Very important)

Type of training	0	1	2	3	4	5	6	7	8	9	10	Total
Introduction and Application of other health research areas	0	0	0	0	0	1	5	9	33	70	94	212
Research methodology	0	0	0	0	0	3	4	11	37	67	90	212
Systematic Reviews	0	0	0	0	2	3	4	15	43	54	91	212
Meta-analysis	0	0	0	0	0	3	7	25	54	57	66	212
Measuring Health Outcomes	0	0	0	0	0	0	7	20	47	61	77	212
Scientific writing	0	0	0	1	0	5	9	12	44	68	73	212
Policy brief writing	0	0	0	0	1	1	7	18	32	71	82	212
Other	0	8	0	0	0	0	0	0	0	0	0	8

Section IV: Opportunities for your organization with HTA and other relevant health evidence

1. Please indicate the type of your organization (select one):

Organisation type	Number
Government organizations (ministries, independent institutions, etc.)	161
Research Institute	12
Provincial hospitals	1
International non-governmental organization	28
Private sector	4
Multilateral/bilateral cooperation organizations	1
Educational institutions	1
Academic institute	4
Total	212

3. At which level does your organization operate?

Level	Number
National	163
Provincial	24
Organisational	3
University	11
International	9
Institute	1
Hospitals	1
Total	212

4. Do you see your organization as a generator or user of HTA evidence?

Type	Number
Generator	24
User	33
Both	153
Other	2
Total	212

8. Please identify criteria for consideration (of

Criteria	Number
High probability of impact of research	158
Capacity to conduct study	111
Feasibility of study within time frame of 1.5 years	97
Other	0

Open-ended questions not summarised here

Section IV

2. Please provide the location (city/town/state) of your organization:

5. Please provide ONE health intervention topic that can potentially be an HTA research topic - *Summarised separately*

6. Please prioritize three most important research topics to inform policy - *Summarised separately*

7. For the priority topic you provided above, please suggest at least ONE research question that you think should be examined. -

9. Please identify your organization's strengths in terms of using and/or generating evidence for HTA and other health research

10. Please identify the constraints your organization faces in terms of using and/or generating evidence for HTA and other health research evidence

11. Do you have any additional comments?
